# Supplementary material for: Identification of coxsackievirus A24 variant during an acute hemorrhagic conjunctivitis outbreak in coastal Kenya, 2024
Source: Wellcome Open Res. 2025 Mar 31;10:28. Originally published 2025 Jan 21. [Version 2] doi: 10.12688/wellcomeopenres.23522.2 (PMC11992516; doi:10.12688/wellcomeopenres.23522.2)
Supplement: Peer review history

# Reviewer report: Reviewer response for version 2

**DOI:** 10.21956/wellcomeopenres.26498.r121519

**Published:** 2025-04-23

**Authors:** Sanjay Tikute

**Affiliations:** 1 ICMR- National Institute of Virology, Pune, India

1. The revised manuscript is checked for the clarifications to the comments raised. Most of them can be accepted. Still in conclusion, CV24v is typed in wrong manner. Please change it as CV-A24v.

 2. In plain language summary, the same mistake is repeated mentioned in point no.1. The manuscript can be accepted ONLY after the above corrections are done.

 Thanks...

Is the work clearly and accurately presented and does it cite the current literature?

Yes

If applicable, is the statistical analysis and its interpretation appropriate?

I cannot comment. A qualified statistician is required.

Are all the source data underlying the results available to ensure full reproducibility?

Yes

Is the study design appropriate and is the work technically sound?

Partly

Are the conclusions drawn adequately supported by the results?

Yes

Are sufficient details of methods and analysis provided to allow replication by others?

Yes

Reviewer Expertise:

Virus isolations, Infectious diseases diagnosis, Viral pahtogenesis, Laboratory animal experimentation.

I confirm that I have read this submission and believe that I have an appropriate level of expertise to confirm that it is of an acceptable scientific standard.

# Reviewer report: Reviewer response for version 2

**DOI:** 10.21956/wellcomeopenres.26498.r121520

**Published:** 2025-04-11

**Authors:** Anchal Thakur

**Affiliations:** 1 Post Graduate Institute of Medical Education and Research, Chandigarh, India

No further comments

Is the work clearly and accurately presented and does it cite the current literature?

Yes

If applicable, is the statistical analysis and its interpretation appropriate?

Partly

Are all the source data underlying the results available to ensure full reproducibility?

Yes

Is the study design appropriate and is the work technically sound?

Yes

Are the conclusions drawn adequately supported by the results?

Yes

Are sufficient details of methods and analysis provided to allow replication by others?

Partly

Reviewer Expertise:

Conjunctivitis, Cataract, Iol, quality of vision

I confirm that I have read this submission and believe that I have an appropriate level of expertise to confirm that it is of an acceptable scientific standard.

# Reviewer report: Reviewer response for version 1

**DOI:** 10.21956/wellcomeopenres.25943.r118288

**Published:** 2025-02-26

**Authors:** Savitri Sharma

**Affiliations:** 1 Jhaveri Microbiology Center, Brien Holden Eye Research Centre (BHERC), L V Prasad Eye Institute (LVPEI), Hyderabad, Telangana, India

This study identified the cause of acute hemorrhagic conjunctivitis in 13 patients using meta genomic platform. Bacterial culture and real time PCR for adenovirus were also done. The methodology is described well for most part except for information on sample collection. 

 1. Please clarify whether different swabs were collected for different tests. It would be good to describe the sample collection method for each of the tests.

 2. **Methods-Bacterial culture:** The method adopted is inadequate in terms of incubation time which should be at least 48 hours. The method of identification of the bacteria has not been given. This lacunae may be noted as a limitation.

 3. Clinical data is completely missing. The number being small it should have been possible to describe the clinical features along with representative figures. Stage of the disease might have been variable in the patients that may explain why all cases were not positive for the virus. 

 The manuscript is well written.

Is the work clearly and accurately presented and does it cite the current literature?

Yes

If applicable, is the statistical analysis and its interpretation appropriate?

Yes

Are all the source data underlying the results available to ensure full reproducibility?

Yes

Is the study design appropriate and is the work technically sound?

Yes

Are the conclusions drawn adequately supported by the results?

Yes

Are sufficient details of methods and analysis provided to allow replication by others?

No

Reviewer Expertise:

clinical ocular  microbiology. Research in fungal keratitis, viral keratitis, microsporidial keratitis, endophthalmitis, Acanthamoeba keratitis etc.

I confirm that I have read this submission and believe that I have an appropriate level of expertise to confirm that it is of an acceptable scientific standard, however I have significant reservations, as outlined above.

# Response

**Published:** 2025-03-21

**Authors:** Arnold Wasike Lambisia

**Affiliations:** Kenya Medical Research Institute, Kenya

1. Please clarify whether different swabs were collected for different tests. It would be good to describe the sample collection method for each of the tests.  

 Our response: Thank you for the comment. We clarify that only ocular swabs were collected and these were used for all the laboratory analysis described; for cultures and nucleic acid analysis approaches that were used for the different molecular tests in the article.

 2. **Methods-Bacterial culture:** The method adopted is inadequate in terms of incubation time which should be at least 48 hours. The method of identification of the bacteria has not been given. This lacunae may be noted as a limitation.  

 Our response: An incubation time of 18-24 hours was sufficient to identify the suspected pathogens and other bacteria known to cause “red eye” e.g., Staphylococcus and Streptococcus. The method of identification the bacteria is added in the methods.

 3. Clinical data is completely missing. The number being small it should have been possible to describe the clinical features along with representative figures. Stage of the disease might have been variable in the patients that may explain why all cases were not positive for the virus.   

 Our response: These were outbreak samples collected at one timepoint i.e when the individuals visited the clinic with “red eye” as the symptom no further clinicla data was provided to describe what stage of the infection the patients were in. We appreciate that this wa one of the limitations of the article and we follow up work with larger sample size underway.

 The manuscript is well written.  Our response: Thank you for a positive view of our work.

# Reviewer report: Reviewer response for version 1

**DOI:** 10.21956/wellcomeopenres.25943.r118289

**Published:** 2025-02-24

**Authors:** Sanjay Tikute

**Affiliations:** 1 ICMR- National Institute of Virology, Pune, India

**Title: Identification of coxsackievirus A24 variant during an acute hemorrhagic conjunctivitis outbreak in coastal Kenya, 2024.**

**General Comments: (Need to revise the manuscript before indexing).**

 1. In abstract (result section) coxsackievirus A24 is written in short form as ‘CV24v’. Please change it and rewrite as “CV-A24v”. Also correct it in entire article.

 2. The outbreak was in the coastal area of Kenya. Therefore more population should be considered for the study. Thirteen patients is a very low number of study subjects.

 3. Clinical details or symptomatic conditions of all the patients should be included.

 4. In background after the word blurred vision the sign ‘=’ is added, please remove it.

 5. In study population paragraph, it is mentioned that after sample collection it was transported to KWTRP at ambient temperature which is not advisable.

 6.  Metagenomic screening paragraph, the word ‘incubating’ is written as ‘Incubating’ remove capital and small one.

 7. In abstract, in the result section, it is mentioned that VP4/2 junction, please explain.

 8. In metagenomics sequencing paragraph, it is mentioned that 3 samples mapped to RVA. After reanalyzing with specific primers all the samples were RVA negative. Add about the primers used and explain about negative results.

 9. The Ct values of the 3 samples ranged between 32.29 to 37.65, what was the positive control and its value.

 10. In table number 1, it is shown that only 2 samples that is , sample 3 and sample 4 after blast hits to rotavirus. What about the third sample previously mentioned. Needs to specify.

**.**

****

Is the work clearly and accurately presented and does it cite the current literature?

Yes

If applicable, is the statistical analysis and its interpretation appropriate?

I cannot comment. A qualified statistician is required.

Are all the source data underlying the results available to ensure full reproducibility?

Yes

Is the study design appropriate and is the work technically sound?

Partly

Are the conclusions drawn adequately supported by the results?

Yes

Are sufficient details of methods and analysis provided to allow replication by others?

Yes

Reviewer Expertise:

Virus isolations, Infectious diseases diagnosis, Viral pahtogenesis, Laboratory animal experimentation.

I confirm that I have read this submission and believe that I have an appropriate level of expertise to confirm that it is of an acceptable scientific standard, however I have significant reservations, as outlined above.

# Response

**Published:** 2025-03-21

**Authors:** Arnold Wasike Lambisia

**Affiliations:** Kenya Medical Research Institute, Kenya

1. In abstract (result section) coxsackievirus A24 is written in short form as ‘CV24v’. Please change it and rewrite as “CV-A24v”. Also correct it in entire article. Our response: Thanks – This is now corrected

 2. The outbreak was in the coastal area of Kenya. Therefore more population should be considered for the study. Thirteen patients is a very low number of study subjects. Our response: The samples were obtianed as part of an outbreak response initiative by the ministry of health. This was not a research study. In such scenarios a few purposively selected samples can be adequate to tease out the cause of the outbreak disease. This convience sampling appraoch is a limitation of the analysis we present. Indeed we have currently a larger molecular epidemiological study underway that will reinforce out findings here.

 3. Clinical details or symptomatic conditions of all the patients should be included. Our response: Unfortunately more detailed symptom data of the cases we analysed are not available to us. As previously indicated , the samples were collected for outbreak response to determine the aetiology of AHC epidemic.The only symptom recorded from the patients was having a  "red eye"/ AHC.

 4. In background after the word blurred vision the sign ‘=’ is added, please remove it. Our response: This has been removed

 5. In study population paragraph, it is mentioned that after sample collection it was transported to KWTRP at ambient temperature which is not advisable. Our response: We agreed that this is not advisable. This may have affected the intergrity of the samples and moving forward we stress cold chain transport.

 6.  Metagenomic screening paragraph, the word ‘incubating’ is written as ‘Incubating’ remove capital and small one. Our response: This has been corrected

 7. In abstract, in the result section, it is mentioned that VP4/2 junction, please explain. Our response: The polyprotein has multiple gens which include the VP4 and VP2 so the primers target the VP4/VP2 junction.

 8. In metagenomics sequencing paragraph, it is mentioned that 3 samples mapped to RVA. After reanalyzing with specific primers all the samples were RVA negative. Add about the primers used and explain about negative results.   Our response: The primers have been added to the methods section. Since the rotavirus reads in the metagenomic sequence output were very few and this was nt confirmed on PCR processing. We assume this may have been contaminants in the samples perharps in the sample processing steps.

 9. The Ct values of the 3 samples ranged between 32.29 to 37.65, what was the positive control and its value. Our response: We did not have a positive control for CV-A24v at the time of running the assay. This was not a commercial kit.

 10. In table number 1, it is shown that only 2 samples that is , sample 3 and sample 4 after blast hits to rotavirus. What about the third sample previously mentioned. Needs to specify.   Our response: That was sample 13 shown in table 1

# Reviewer report: Reviewer response for version 1

**DOI:** 10.21956/wellcomeopenres.25943.r118292

**Published:** 2025-02-24

**Authors:** Milton Mogotsi

**Affiliations:** 1 Next Generation Sequencing Unit and Division of Virology, Faculty of Health Sciences, University of the Free State, Bloemfontein, South Africa

**Review Report:**

**Title: Identification of coxsackievirus A24 variant during an acute hemorrhagic conjunctivitis outbreak in coastal Kenya, 2024.**

 The study by Lambisia and colleagues investigated the aetiology of an acute haemorrhagic conjunctivitis (AHC) outbreak in Kenya's coastal region in 2024. Utilizing metagenomic sequencing, the authors identified Coxsackievirus A24 variant (CA24v) as the potential causative agent, marking the first confirmed detection of CA24v in Kenyan AHC cases. Phylogenetic analysis placed the Kenyan strains within genotype IV (GIV), consistent with recent global outbreaks. The findings underscore the utility of metagenomic sequencing in identifying pathogens in new epidemics and highlight the need for enhanced diagnostic capacities in outbreak settings.

**Comments:**

 The study addresses a significant public health concern by identifying the potential causative agent of a recent AHC outbreak in Kenya, providing critical information for public health responses. Metagenomic sequencing allowed for the detection of CA24v, a pathogen not typically identified through conventional diagnostic methods. On the other hand, phylogenetic analysis offered valuable insights into the genetic relationship of the Kenyan strains with global CA24v variants, contributing to the understanding of the virus's epidemiology. However, the study's conclusions are based on a small number of samples from a single geographic region, which may not fully represent the broader epidemiological landscape of AHC in Kenya. Additionally, the absence of detailed patient demographics and clinical data limits the ability to assess the full impact of the outbreak and the virus's behaviour in different populations. Furthermore, the study relies on samples from symptomatic cases, which may not capture the full spectrum of infection, including asymptomatic or mild cases.

 Moreover, I have a concern regarding the absence of a positive control especially in the real-time PCR assay for adenovirus detection. We cannot conclude with certainty that the samples were negative for adenovirus when there was no positive control included. The negative results could be a results of PCR conditions, or issues with the primers, for instance. Also, the authors performed DNase treatment to digest all DNA in the samples prior cDNA and metagenomic sequencing. However, I am of the opinion that the authors should have processed the samples in parallel (DNase-treated and DNase-non treated) to confirm if indeed the samples were adenovirus negative considering that it is a DNA virus and it might have been removed by nuclease digestion.

 My recommendations regarding this paper are that future studies should include a larger and more diverse sample set from multiple regions to provide a more comprehensive understanding of the outbreak's scope and the virus's epidemiology. Authors need to also incorporate detailed patient demographics, and clinical presentations which will be useful in assessing the impact of the outbreak and inform targeted public health interventions.

 I concur with the authors that long-term surveillance and studies are necessary to monitor the persistence and evolution of CA24v in the region, as well as to evaluate the effectiveness of public health measures implemented in response to the outbreak. While the findings are significant, providing valuable insights into the aetiology of the AHC outbreak in Kenya, I strongly recommend that the authors consider publishing these results as a brief report instead of a full research article due to the above-mentioned limitations such as small sample size and lack of demographic/clinical data.

Is the work clearly and accurately presented and does it cite the current literature?

Yes

If applicable, is the statistical analysis and its interpretation appropriate?

Not applicable

Are all the source data underlying the results available to ensure full reproducibility?

Yes

Is the study design appropriate and is the work technically sound?

Partly

Are the conclusions drawn adequately supported by the results?

Partly

Are sufficient details of methods and analysis provided to allow replication by others?

Yes

Reviewer Expertise:

Virology, metagenomics, infectious disease

I confirm that I have read this submission and believe that I have an appropriate level of expertise to confirm that it is of an acceptable scientific standard, however I have significant reservations, as outlined above.

# Response

**Published:** 2025-03-21

**Authors:** Arnold Wasike Lambisia

**Affiliations:** Kenya Medical Research Institute, Kenya

**Comments:**

 The study addresses a significant public health concern by identifying the potential causative agent of a recent AHC outbreak in Kenya, providing critical information for public health responses. Metagenomic sequencing allowed for the detection of CA24v, a pathogen not typically identified through conventional diagnostic methods. On the other hand, phylogenetic analysis offered valuable insights into the genetic relationship of the Kenyan strains with global CA24v variants, contributing to the understanding of the virus's epidemiology. **However, the study's conclusions are based on a small number of samples from a single geographic region, which may not fully represent the broader epidemiological landscape of AHC in Kenya**. Additionally, the **absence of detailed patient demographics and clinical data limits** the ability to assess the full impact of the outbreak and the virus's behaviour in different populations. Furthermore, the study relies on samples from symptomatic cases, which may not capture the full spectrum of infection, including asymptomatic or mild cases.   Our response: Thanks for these comments. We agree that our study is indeed limited by the small number samples analysed coupled to lack of detailed clinical data. This to some extent limits our conclusions. However de do still think this analysus gives us some important first insight on the likely cause of the AHC outbreak we recorded. Follow up bigger and carefully designed epidemiological study analysing samples collected over a broader geographical area and from individiduals with and without these symptoms or of different clinical severity will be an important follow up to our findings here. Indeed we are already currently leveraging a study that was going on in the community at this time of the outbreak that collected serial samples and detailed clinical data from the same individual and ongoing analysis will identify the role of the virus in symptomatic and asymptomatic individuals and other potential pathogens involved in AHC.

 Moreover, I have a concern regarding the absence of a positive control especially in the real-time PCR assay for adenovirus detection. We cannot conclude with certainty that the samples were negative for adenovirus when there was no positive control included. The negative results could be a results of PCR conditions, or issues with the primers, for instance. Also, the authors performed DNase treatment to digest all DNA in the samples prior cDNA and metagenomic sequencing. However, I am of the opinion that the authors should have processed the samples in parallel (DNase-treated and DNase-non treated) to confirm if indeed the samples were adenovirus negative considering that it is a DNA virus and it might have been removed by nuclease digestion.   Our response: The adenovirus real-time PCR had a negative and positive control and these performed well. A statement has been added for the same. The metagenomics protocol may have missed any additional DNA virus but if it was actively replicating there is a good chance of detecting it. Actually our previous metagenomic work despite DNAase digestion we detected a number of DNA viruses e.g. bocavirus, herpesvirus 1 and 5, see in https://www.nature.com/articles/s41598-021-03987-1. Our assay has still a good chance of detecting DNA virus, probably because of their transcriptomes. None-the less this is still a nice suggestion for taking DNase-treated and DNase-non treated approach for future work although the latter has challange of alot of host DNA contamination.

 My recommendations regarding this paper are that future studies should include a larger and more diverse sample set from multiple regions to provide a more comprehensive understanding of the outbreak's scope and the virus's epidemiology. Authors need to also incorporate detailed patient demographics, and clinical presentations which will be useful in assessing the impact of the outbreak and inform targeted public health interventions.   Our response: We agree. Indeed we how have a study underway looking at a well followed cohort over time is currently evaluating the extent of CA24v locally during the same period and this will provide a much clearer picture on the impact of the outbreak in the community

 I concur with the authors that long-term surveillance and studies are necessary to monitor the persistence and evolution of CA24v in the region, as well as to evaluate the effectiveness of public health measures implemented in response to the outbreak. While the findings are significant, providing valuable insights into the aetiology of the AHC outbreak in Kenya, I strongly recommend that the authors consider publishing these results as a brief report instead of a full research article due to the above-mentioned limitations such as small sample size and lack of demographic/clinical data.   Our response: Many thanks for the suggestion. We did consider this but the challange is that such a report ends up even trimming further the details we have provided which are already considered as limited and more information has been requested. Here we have really attempted providing all details that were within our disposal to enhance readers understanding.

# Reviewer report: Reviewer response for version 1

**DOI:** 10.21956/wellcomeopenres.25943.r117662

**Published:** 2025-02-21

**Authors:** Anchal Thakur

**Affiliations:** 1 Post Graduate Institute of Medical Education and Research, Chandigarh, India

**Here are few comments from my side:**

 1. The authors have mentioned that quantitative PCR was used for CVA24v detection but there is no mention of viral load in the results section. Please take a look at the MIQE guidelines for viral load results. 

 2. Methodology for Rotavirus group A specific quantitative PCR not mentioned.

 3. Fluorophore and quencher for probe used in CA24v specific qPCR targetting 5'UTR region  is not mentioned.

 3. Typographical errors: "conjuctivitis" in place of conjunctivitis

 4.  The line "All the 13 samples were reanalysed using a CA24v and RVA specific quantitative PCR targeting the NSP3 genome segment"  should be reframed as NSP3 in not present in CA24v and thus can create confusion among readers. 

 the sample size is too small

Is the work clearly and accurately presented and does it cite the current literature?

Yes

If applicable, is the statistical analysis and its interpretation appropriate?

Partly

Are all the source data underlying the results available to ensure full reproducibility?

Yes

Is the study design appropriate and is the work technically sound?

Yes

Are the conclusions drawn adequately supported by the results?

Yes

Are sufficient details of methods and analysis provided to allow replication by others?

Partly

Reviewer Expertise:

Conjunctivitis, Cataract, Iol, quality of vision

I confirm that I have read this submission and believe that I have an appropriate level of expertise to confirm that it is of an acceptable scientific standard, however I have significant reservations, as outlined above.

# Response

**Published:** 2025-03-21

**Authors:** Arnold Wasike Lambisia

**Affiliations:** Kenya Medical Research Institute, Kenya

The authors have mentioned that quantitative PCR was used for CVA24v detection but there is no mention of viral load in the results section. Please take a look at the MIQE guidelines for viral load results.    Our response?

 2. Methodology for Rotavirus group A specific quantitative PCR not mentioned.   Our response: Thanks for highlighting this. This is now added.

 3. Fluorophore and quencher for probe used in CA24v specific qPCR targetting 5'UTR region  is not mentioned.   Our response: Thanks for highlighting. This is now added.

 3. Typographical errors: "conjuctivitis" in place of conjunctivitis   Our response: This has been corrected on the references

 4.  The line "All the 13 samples were reanalysed using a CA24v and RVA specific quantitative PCR targeting the NSP3 genome segment"  should be reframed as NSP3 in not present in CA24v and thus can create confusion among readers.    Our response: The latter part on NSP3 has been deleted to avoid confusion and stated in the methods.

 5. the sample size is too small Our response: This is a limitation of this current analysis, which is preliminary. We have since learnt that the virus can be detected in respiratory samples and were are currently working on  >100 samples from individuals who attended an oupatient clinic during an outbreak and had symptoms and the virus detected. This bigger analysis will be presented in due course to understand this virus better.
